# 193. Impact of COVID-19 Pandemic on Pulmonary Tuberculosis Evaluations and Diagnosis at a Large Safety Net Hospital in Los Angeles, California

**DOI:** 10.1093/ofid/ofab466.193

**Published:** 2021-12-04

**Authors:** Aditya J Jones, Edward C Jones-Lopez, Susan Bulter-Wu, Melissa L Wilson, John Rodman, Christian Voyageur, Brenda E Jones

**Affiliations:** 1 Los Angeles County + University of Southern California (LAC +USC) Medical Center, Los Angeles, California; 2 Keck School of Medicine University of Southern California, Los Angeles, California; 3 Keck School of Medicine, University of Southern California, Los Angeles, CA; 4 USC School of Medicine, Los Angeles, California; 5 USC, Los Angeles, California; 6 Los Angeles County and University of Southern California (LAC +USC) Medical Center, Los Angeles, California; 7 University of Southern California, Pasadena, California

## Abstract

**Background:**

There is significant global concern that the COVID-19 pandemic may negatively impact tuberculosis (TB) control. This is a descriptive analysis of TB evaluations and diagnosis during 2019 (pre COVID-19 period) and 2020 (COVID-19 period) at the largest safety net hospital in Los Angeles County (LAC+USC Medical Center).

**Methods:**

The medical records of patients diagnosed with pulmonary TB from January 1, 2019 to December 31, 2020 were identified through laboratory and electronic medical records. We included all patients with ≥ 1 sputum positive result for Mycobacterium tuberculosis (MTB) culture and reviewed their Xpert MTB/RIF MTB PCR.

**Results:**

Table 1 shows summary of results. During the COVID-19 period, the number of patients evaluated for pulmonary TB decreased by 64% compared to the previous year (Figure 1). The proportion of patients with culture-confirmed TB disease however, was nearly identical (P=0.913) (Table 1). Sputum acid-fast bacilli (AFB) smear positivity increased 52% to 64% during COVID-19 (P=0.324) and disease severity as measured by chest radiograph, was significantly higher during the COVID-19 period (P = 0.031) (Figure 2).

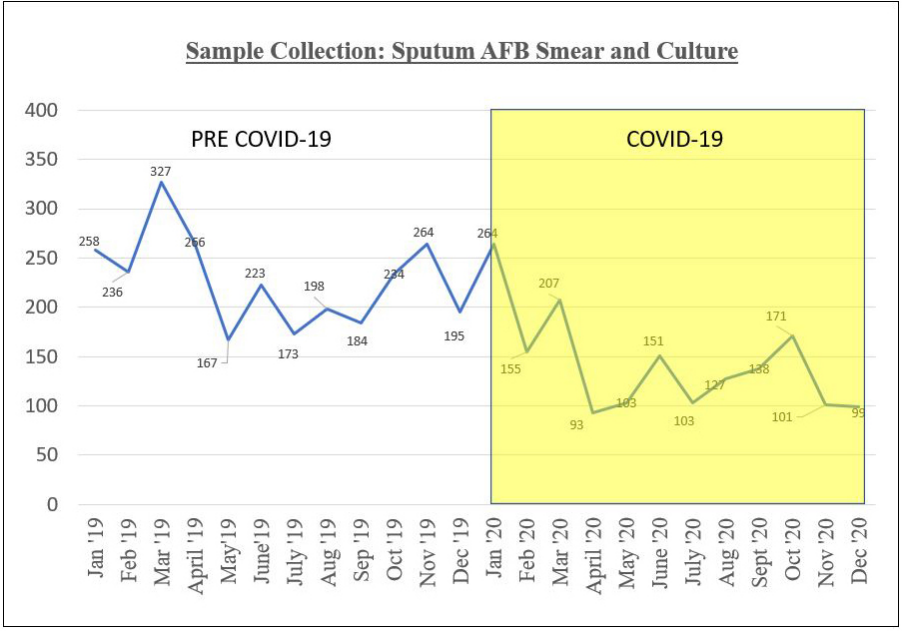

Trend of sputum AFB smear and culture samples collected from January 1, 2019 to December 31, 2020.

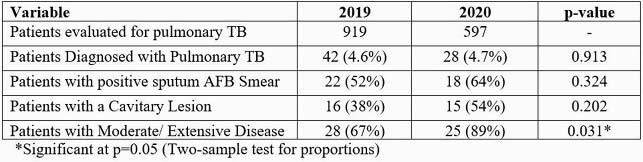

Summary of results of patients diagnosed with pulmonary TB from January 1, 2019 to December 31, 2020 at LAC+USC Medical Center.

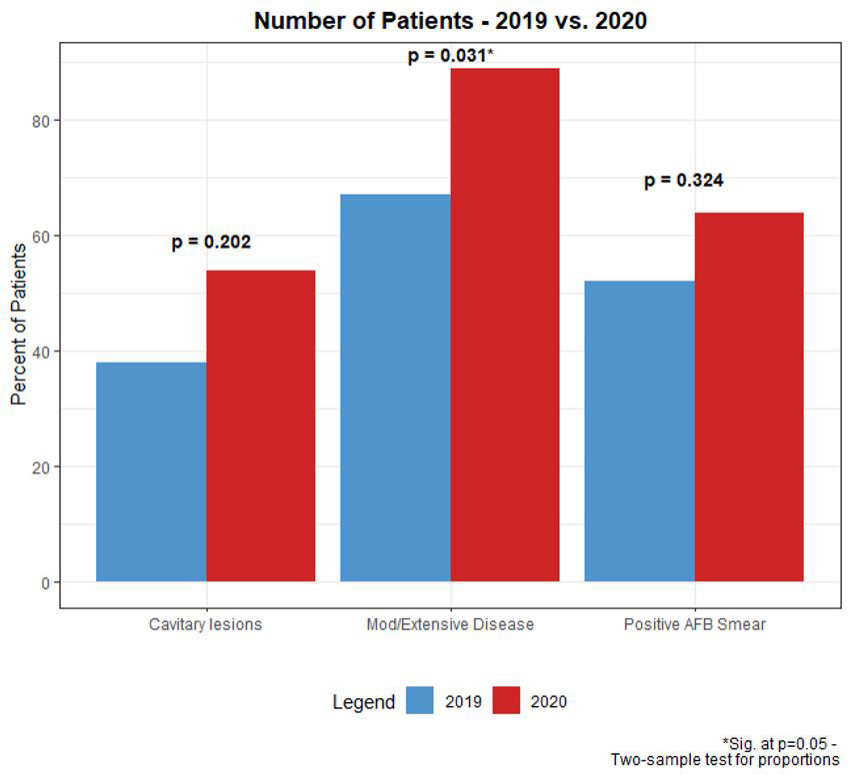

Results of two-sample test for proportions of 2019 vs 2020 for cavitary lesions, extent of disease, and sputum positive AFB smear microscopy.

**Conclusion:**

These preliminary results suggest that when compared to the previous year, the number of pulmonary TB evaluations decreased by 64% during the COVID period. Whereas the proportion of patients diagnosed with TB disease was similar, TB patients during the COVID-19 period had more advanced disease at diagnosis, as measured by sputum smear AFB microscopy and disease severity on chest radiograph (P=0.031). These data suggest potentially consequential interruptions and delays in pulmonary TB diagnosis during the COVID-19 period.

**Disclosures:**

**Susan Bulter-Wu, PhD** , **Cepheid** (Consultant)

